# WNT5A in Cancer: A Pan-Cancer Analysis Revealing Its Diagnostic and Prognostic Biomarker Potential

**DOI:** 10.7759/cureus.65190

**Published:** 2024-07-23

**Authors:** Mutaz Mohammed Abdallah, Mawada Yahia, Yousra Tagelsir Ahmed, Mohamed Alfaki

**Affiliations:** 1 Microbiology, College of Life Sciences, Northeast Forestry University, Harbin, CHN; 2 Medical Laboratory Science/Medical Microbiology, University of Khartoum, Khartoum, SDN; 3 Microbiology, Faculty of Medical Laboratory Science, Alzaiem Alazhari University, Khartoum, SDN; 4 Research, Sidra Medicine, Doha, QAT

**Keywords:** genetic oncology, lung, cancer-genetics, genetics, bioinformatics

## Abstract

Background and objective: The wingless-related integration site family (WNT) signaling pathway is critical for tumor progression and development. It is associated with various neoplasms produced by WNT pathway deregulation; WNT5A, a member of the WNT family, has been linked to carcinogenesis, exhibiting either oncogenic or tumor-suppressive effects. The study investigates how the gene affects certain types of cancer. The study aimed to evaluate the potential prognostic significance of WNT5A genes as diagnostic biomarkers for various types of cancer.

Methodology: We investigated WNT5A gene expression in a pan-cancer analysis using various bioinformatics databases, including GEPIA (Gene Expression Profiling Interactive Analysis), TIMER2 (Tumor Immune Estimation Resource, Version 2), the University of Alabama at Birmingham Cancer Data Analysis Portal UACLAN databases, the Kaplan-Meier (K-M) plotter, cBioPortal, and Gene Set Cancer Analysis (GSCA).

We aimed to gain insight into the expression of WNT5A in various tumors and its relationship with immune infiltration, overall survival, and genetic changes. Public datasets validated WNT5A expression in lung squamous cell carcinoma (LUSC) and stomach adenocarcinoma (STAD) samples.

Results: WNT5A pan-cancer analysis was highly expressed in two cancer types, including STAD and LUSC. Additionally, TIMER results showed a positive association of WNT5A with immune cell infiltration in LUSC and STAD. Survival analysis indicated that LUSC cancer exhibits better overall survival, while STAD has lower overall survival levels, which means a poor prognosis in the STAD cancer type. Furthermore, mutation analysis revealed that the WNT5A gene was mutated in 1.4% of cases, with most alterations being deletions followed by amplifications.

Conclusions: The WNT5A gene's high expression in many malignancies, including LUSC and STAD, suggests it could be used as a diagnostic biomarker. This study shows a relationship between WNT5A expression and immune cell abundance in LUSC and STAD. Our pan-cancer analysis of this gene is the first of its type, and it will inform future research, comprehensive investigation, and wet lab experiments.

## Introduction

According to the World Health Organization (WHO), cancer is a leading cause of death worldwide, accounting for nearly 10 million deaths in 2020, or nearly one in six deaths. The most common cancers are breast, lung, colon, rectum, and prostate cancers. All cancers are linked with many genes, like the wingless gene family. This wingless-related integration site family (WNT) signaling is crucial in the progression and development of tumors. It is associated with many neoplasms caused by the deregulation of the WNT pathway [1]. A total of 19 WNT members have been identified in mammals. These include WNT1, WNT2, WNT2b/13, WNT3, WNT3a, WNT4, WNT5A, WNT5b, WNT6, WNT7a, WNT7b, WNT8a/d, WNT8b, WNT10a, WNT10b/12, WNT11, WNT14, WNT15, and WNT16 [2]. WNT5A, one member of the WNT family, has been linked to carcinogenesis, displaying either oncogenic or tumor-suppressive properties. In contrast, the expression of it promotes the aggressiveness of tumors by stimulating the invasion and spread of cancer cells. Interestingly, WNT5A may also have tumor-suppressive effects by opposing the canonical Wnt pathway, which inhibits cell growth and migration. Its involvement in colorectal cancer, where it is downregulated, as well as in other cancers such as lung, gastric, prostate, melanoma, and ovarian cancer, where it can be upregulated and play a role in tumor development and progression [3-5]. The role of WNT5A in different cancer types is complex and not fully understood, with evidence of both tumor-suppressing and tumor-promoting effects. Therefore, the ongoing study aims to clarify its precise molecular role in cancer by using bioinformatics tools and datasets, such as GEPIA, UACLAN, and TIMER2, which significantly impact cancer detection. It will also explore gene expression, immune infiltration, and patient prognosis in different types of cancer and treatment [6-7].

The study objective was to evaluate the potential prognostic significance of WNT5A genes as diagnostic biomarkers for various types of cancer, such as glioblastoma (GMB), lung squamous cell carcinoma (LUSC), and lung adenocarcinoma (LUAD).

## Materials and methods

Knowledge gap assessment 

The query step involves searching the literature for current information on the role of WNT5A. The query was searched in the PubMed database to generate a PubMed query that collected the pool of publications that comprise the WNT5A literature. The query includes all official names, symbols, and aliases listed for WNT5A in the Swissprot, OMIM, and Entrez gene databases from the National Cancer Institute. The [tw] parameter is added to make a PubMed search as instructed. [(tw) = text words: this limits the search to text found in tiles and abstracts]. WNT5A[tw] OR "Wnt family member 5A"[tw] OR "WNT-5A protein"[tw] OR "epididymis secretory sperm binding protein"[tw] OR "protein Wnt-5a"[tw] OR "wingless-type MMTV integration site family, member 5A"[tw] OR "hWNT5A"[tw] and GEPIA OR UACLAN.

The Gene Expression Profiling Interactive Analysis (GEPIA) analysis

GEPIA (http://gepia.cancer-pku.cn/index.html ) is a web-based tool that offers rapid and flexible features derived from The Cancer Genome Atlas (TCGA) and Genotype-Tissue Expression (GTEx) project. It provides crucial interactive functions such as differential expression analysis, profiling plotting, correlation analysis, and patient survival analysis [8]. We used the GEPIA tool to examine the WNT5A expression levels in tumor samples and their corresponding normal tissues.

Gene expression analysis with TIMER

The expression levels of WNT5A genes in various forms of cancer were assessed using the TIMER (Tumor Immune Estimation Resource) databases available at http://timer.comp-genomics.org/. TIMER is an accessible online resource that provides information on 32 different forms of cancer. It contains 10,897 samples from TCGA database [9].

UALCAN database analysis 

UALCAN is a simple-to-use and interactive online platform, accessible at http://ualcan.path.uab.edu, that enables comprehensive analysis of TCGA gene expression data [10]. The UALCAN dataset has been used to perform the subgroup analysis and conduct a more in-depth examination of the variables that influence the expression of WNT5A. We applied UALCAN to validate the expression data of WNT5A by analyzing it through the *Expression Analysis* module and the TCGA dataset.

Kaplan-Meier (K-M) plotter analysis

The K-M plotter is a tool that can analyze the impact of 54,000 genes (mRNA, microRNA, and protein) on survival rates in 21 cancer types, such as breast, ovarian, lung, and GC. The databases used to source the data include GEO, EGA, and TCGA [11, 12]. The K-M plot recognizes 70,632 gene symbols, including HUGO Gene Nomenclature Committee-approved official gene symbols, previous symbols, and aliases. The tool is primarily used for discovering and validating survival biomarkers through meta-analysis. We utilized a K-M plotter to confirm the prognostic values of WNT5A and the significantly correlated genes in different cancers.

GEPIA survival analysis

We used the Survival Analysis module of GEPIA to obtain overall survival (OS) data for WNT5A genes in STAD, and LUSC based on TCGA [8]. A cutoff value of 50% was set as the threshold to separate high- and low-expression cohorts. The log-rank test was used, and statistical significance was considered at *P*-value < 0.05.

Gene mutation analysis

Investigating the mutation types, mutated location, and structure of WNT5A using cBioPortal. The cBioPortal for Cancer Genomics (http://cbioportal.org ) is a website that offers a platform for studying, displaying, and analyzing complex cancer genomics data [13]. We used cBioPortal to investigate the aberration subtypes of subgroups and the location and structure of WTN5A in GC. WNT5A mutation analysis used the cBioPortal database to calculate the mutation frequency.

Mutation correlation analysis using GSCA databases

The analysis of WNT5A mutation and calculation of correlations between CNV, SNV, and other cancers was done by using Gene Set Cancer Analysis (GSCA) [3-6]. GSCA (http://bioinfo.life.hust.edu.cn/GSCA) [8] is used to analyze cancer gene sets at the genomic, pharmacological, and immune genomic levels and investigate the links between immune infiltration, gene expression, genomic variation, gene set expression/mutation, and clinical outcome [14].

TIMER analysis

The TIMER web server, accessible via URL (https://cistrome.shinyapps.io/timer/), is a comprehensive resource for analyzing immune infiltrates across various cancer types in a systematic manner. The TIMER algorithm estimates the abundances of six different types of immune infiltrates, including B cells, CD4+ T cells, CD8+ T cells, neutrophils, macrophages, and dendritic cells. Users can input function-specific parameters on the web server, and the resulting figures are dynamically displayed to enable convenient access to tumor immunological, clinical, and genomic features [11,12]. The TIMER dataset was used to investigate the link between immune infiltration and prognosis, the link between immune cell infiltration and WNT5A, and the connection between immune infiltration and outcomes for different types of cancer.

## Results

WNT5A pan-cancer analysis

**Figure 1:**
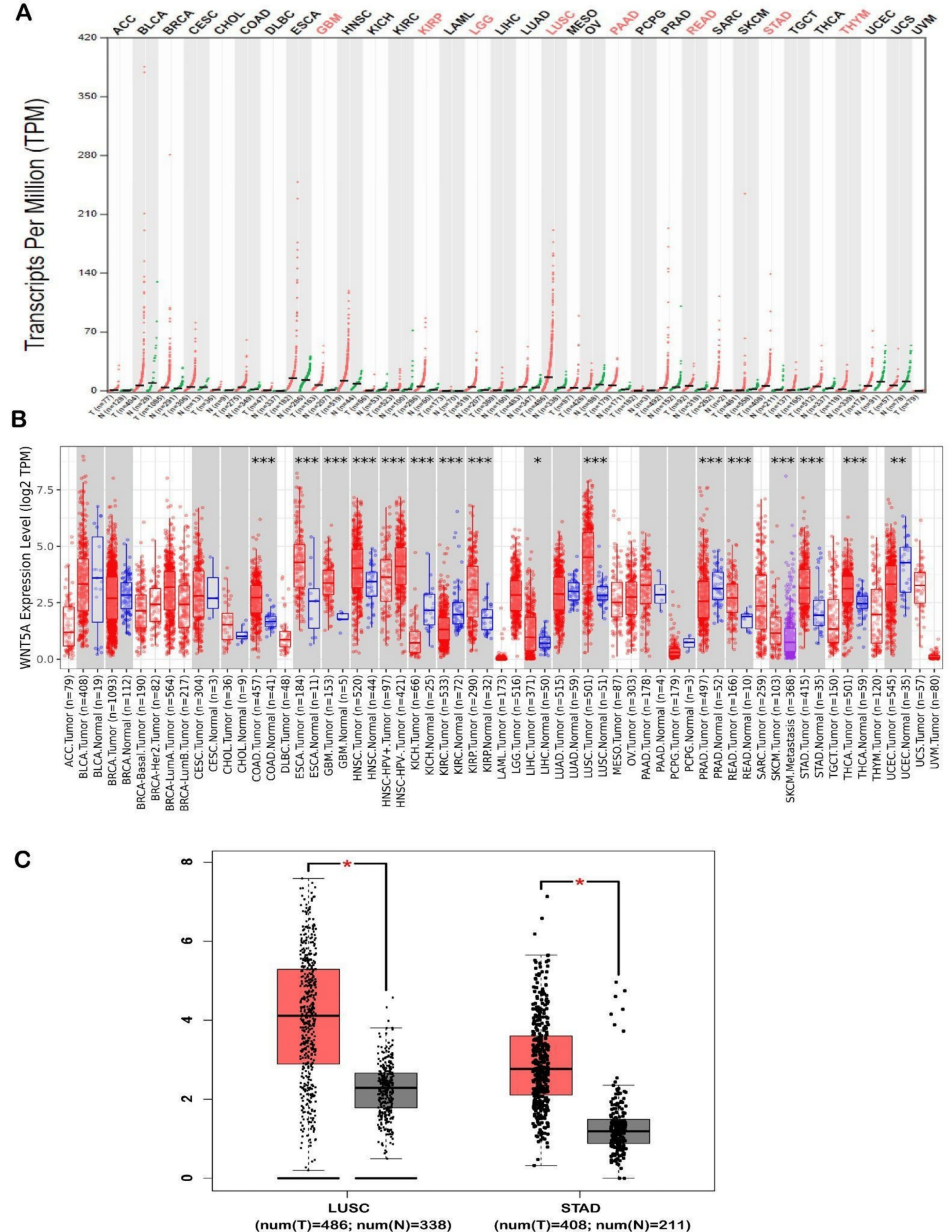
The gene expression profile of the WNT5A gene. The gene expression profile of the WNT5A gene across all tumor samples and paired normal tissues using (A) GEPIA and (B) TIMER databases. Dot plot: Each dot represents an expression of samples. (C) Analysis of LUSC and STAD cancer types between WNT5A genes and their expression between TCGA tumor tissues and the GTEx database of the corresponding normal tissues. *P < 0.05. **P < 0.01. ***P < 0.001. ****P < 0.0001. GEPIA, Gene Expression Profiling Interactive Analysis; TIMER, Tumor Immune Estimation Resource; LUSC, lung squamous cell carcinoma; STAD, stomach adenocarcinoma; TCGA, The Cancer Genome Atlas

The analysis of WNT5A expression in different tumor types was conducted using the GEPIA and TIMER public databases. The utilization of the analysis found that WNT5A expression was significantly upregulated in eight cancers shown by GEPIA databases. These cancers include glioblastoma multiforme (GBM), kidney renal papillary cell carcinoma brain (KIRP), lower grade glioma (LGG), LUSC, pancreatic adenocarcinoma (PAAD), rectum adenocarcinoma (READ), stomach adenocarcinoma (STAD), and thymoma (THYM). Moreover, the expression of WNT5A significantly increased in two types of cancer (LUSC and STAD) compared to normal samples (Figure 1C). Furthermore, an investigation of WNT5A expression was conducted using TIMER, revealing an upregulation of WNT5A in[MAIA1] nine cancer types, including colon adenocarcinoma (COAD) (num(T) = 457; num(N) = 41), GBM (num(T) = 153; num(N) = 5), head and neck squamous cell carcinoma (HNSC) (num(T) = 520; num(N) = 44), KIRP (num(T) = 290; num(N) = 32), liver hepatocellular carcinoma (LIHC) (num(T) = 371; num(N) = 50), LUSC (num(T) = 501; num(N) = 51), READ (num(T) = 166; num(N) = 10), STAD (num(T) = 412; num(N) = 35), thyroid carcinoma (THCA) (num(T) = 501; num(N) = 59). Additionally, it was shown that the expression of WNT5A in metastatic tissues of skin cutaneous melanoma (SKCM) was more significant than in tumor tissues of SKCM. However, as of March 2024, there is a lack of available data to compare either SKCM tumor or metastasis tissues with normal tissues. Moreover, WNT5A expression showed downregulation in five cancer types, including esophageal carcinoma (ESCA) (num(T) = 184; num(N) = 11), kidney chromophobe (KICH) (num(T) = 66; num(N) = 25), kidney renal clear cell carcinoma (KIRC) (num(T) = 533; num(N) = 72), prostate adenocarcinoma (PRAD) (num(T) = 497; num(N) = 52), and uterine corpus endometrial carcinoma (num(T) = 545; num(N) = 35) (Figure 1B).

WNT5A expression analysis

**Figure 2:**
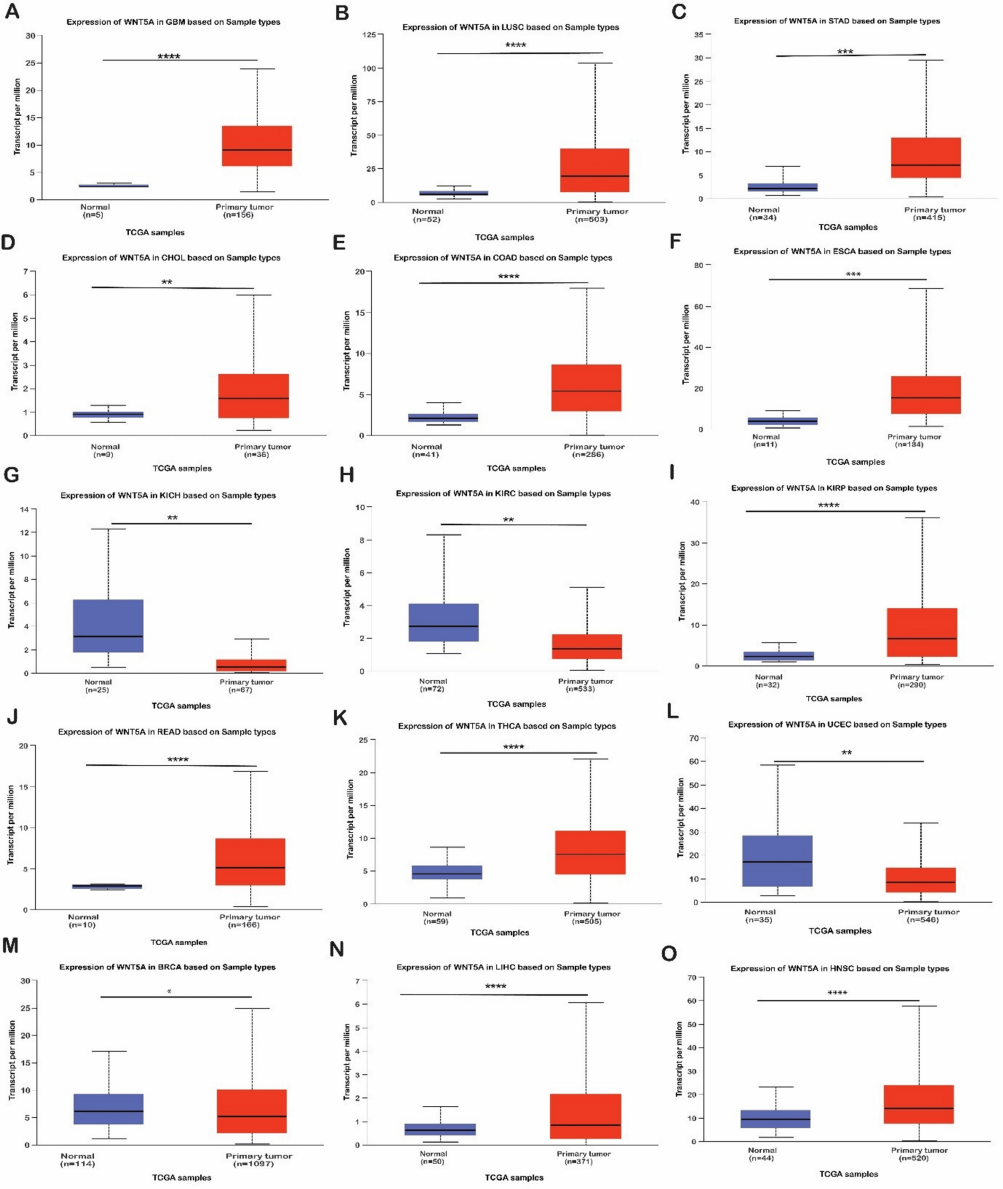
The expression profiles of WNT5A across different cancers using the UALCAN database. The figure depicts the expression profiles of WNT5A across different cancers using the UALCAN database. The boxplot color coding is as follows: blue indicates normal samples, and red indicates tumor samples. The *P*-values are represented as follows: *P < 0.05, **P < 0.01, ***P < 0.001, and ****P < 0.0001.

The UALCAN database was used to validate the expression of WNT5A in 15 cancer samples, which showed significant differences acquired from the GEPIA and TIMER databases. This analysis indicated that WNT5A expression is over-expression in GBM, LUSC, STAD, RBCA, CHOL, COAD, ESCA, HNSC, KIRP, LIHC, READ, and THCN tissues (Figure 2, Tables 1-2). We also discovered that WNT5A mRNA expression was significantly under-expressed in KICH, KIRC, and UCEC tissues compared to normal tissues. Furthermore, we included two cancers (LUSC and STAD) decided upon by the three databases (UALCAN, TIMER, and GEPIA) regarding WNT5A expression for further investigation. 

**Table 1:** The relation between WNT5A expression levels and different clinical features. Represent the expression analysis of WNT5A with LUSC cancer type in different clinical features between cancers and normal samples via the UACLAN database. *P < 0.05. **P < 0.01. ****P < 0.0001. LUSC, lung squamous cell carcinoma

Variable		Low	High	*P*-value	Sign.
Sample types	Normal (*n *= 114)	2.445	11.862	1.11E-16	****
	Primary tumor (*n *= 1,097)	0.151	103.534		
patient's gender	Normal (*n *= 52)	2.445	11.862	-	-
	Male (*n *= 366)	0.221	116.262	<1E-12	****
	Female (*n *= 128)	0.222	76.951	9.14E-11	****
Patient's race	Normal (*n *= 52)	2.445	11.862	-	-
	Caucasian (*n *= 343)	0.221	109.592	<1E-12	****
	African-American (*n *= 30)	0.222	82.308	8.49E-05	****
	Asian (*n *= 9)	5.478	39.514	4.4E-02	*
Patient's age	Normal (*n *= 52)	2.445	11.862	-	-
	21-40 years (*n *= 2)	4.404	5.101	4.07E-01	-
	41-60 years (*n *= 103)	1.215	128.445	1.63E-12	****
	61-80 years (*n *= 361)	0.221	100.984	1.62E-12	****
	81-100 years (*n *= 20)	5.21	41.475	1.55E-02	*
	21-40 years vs. 61-80 years	-	-	<1E-12	****
	21-40 years vs. 81-100 years	-	-	9.56E-03	**
Cancer stages	Normal (*n* = 52)	2.445	11.862	-	-
	Stage 1 (*n* = 243)	0.222	107.692	1.62E-12	****
	Stage 2 (*n* = 157)	0.603	100.671	4.00E-15	****
	Stage 3 (*n* = 85)	0.221	83.526	3.00E-09	****
	Stage 4 (*n* = 7)	1.417	16.866	2.10E-01	-
	Stage 1 vs. Stage 4	-	-	1.38E-03	**
	Stage 2 vs. Stage 4	-	-	2.78E-03	**
	Stage 3 vs. Stage 4	-	-	1.10E-02	*

**Table 2:** The relation between WNT5A expression levels and different clinical features. Represent the expression analysis of WNT5A with STAD cancer type in different clinical features between cancers and normal samples via the UACLAN database. *P < 0.05. **P < 0.01. ***P < 0.001. ****P < 0.0001. STAD, stomach adenocarcinoma

Variable		Low	High	*P*-value	Sign.
Sample types	Normal (n = 34)	0.618	6.911	1.20E-04	***
	Primary tumor (n = 415)	0.405	29.589		
Patient's gender	Normal (n = 34)	0.618	6.911	-	-
	Male (n = 268)	0.405	29.589	1.16E-04	***
	Female (n = 147)	1.445	27.983	6.70E-03	**
Patient's race	Normal (n = 34)	0.618	6.911	-	-
	Caucasian (n = 260)	0.405	29.589	6.00E-04	***
	African-American (n = 12)	2.755	8.814	2.68E-01	-
	Asian (n = 87)	1.174	22.644	2.18E-02	*
Patient's age	Normal (n = 34)	0.618	6.911	-	-
	21-40 years (n = 4)	3.717	8.3	9.24E-01	-
	41-60 years (n = 128)	0.44	26.249	2.39E-02	*
	61-80 years (n = 253)	0.405	30.025	9.74E-05	****
	81-100 years (n = 100)	2.502	33.586	7.00E-03	**
	21-40 years vs. 41-60 years	-	-	3.32E-02	*
	21-40 years vs. 61-80 years	-	-	2.44E-03	**
	21-40 years vs. 81-100 years	-	-	1.46E-02	*
	41-60 years vs. 61-80 years	-	-	4.11E-02	*
Cancer stages	Normal (*n *= 34)	0.618	6.911	-	-
	Stage 1 (*n *= 18)	1.395	19.169	1.18E-01	-
	Stage 2 (*n *= 123)	1.174	28.289	1.38E-03	**
	Stage 3 (*n *= 169)	0.44	26.594	3.58E-04	***
	Stage 4 (*n *= 41)	2.211	33.586	8.95E-04	***

Following thorough data analysis, we found that WNT5A is overexpressed in patients with LUSC and STAD. In LUSC, overexpression was observed in males and females, Caucasians, and African Americans in stages 1, 2, and 3, aged 61 to 80 years (*n* = 361). In STAD, overexpression was noted in male Caucasians in stages 3 and 4, aged 61 to 80 years (*n *= 253). Therefore, WNT5A should be explored as a biomarker or target in LUSC and STAD.

Relationship between WNT5A and survival in pan cancer

K-M plotter was used to analyze the correlation between WNT5A gene expression and the OS (*P* < 0.05) of 21 cancer types.

Prognostic Value of WNT5A in Cancer Type

**Figure 3:**
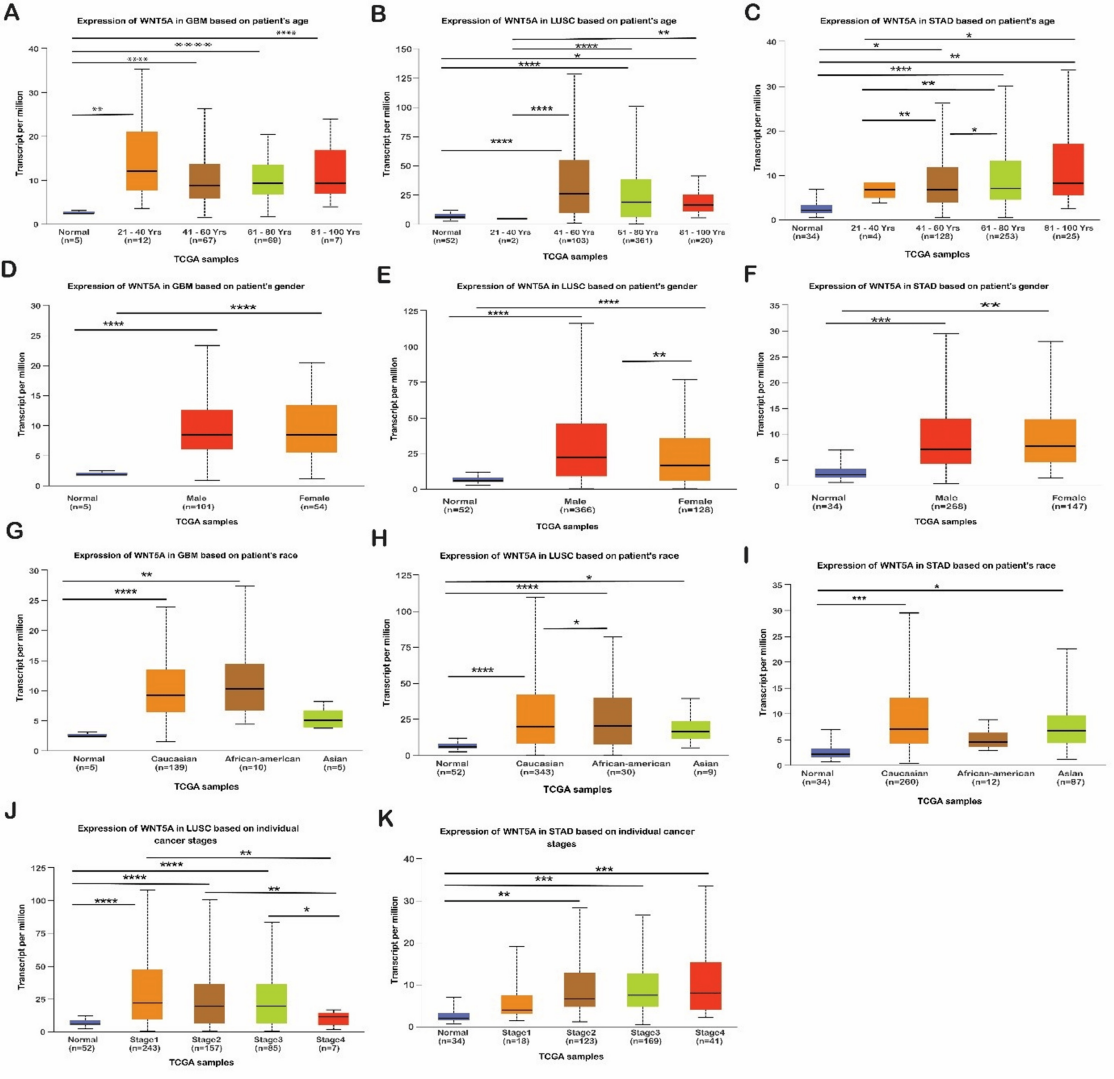
The expression analysis of WNT5A in different clinical features between cancers and normal samples via the UACLAN database. The expression analysis of WNT5A in different clinical features between cancers and normal samples via the UACLAN database. (A-C) The expression status of the WNT5A gene on GBM, LUSC, and STAD cancer is based on the patient’s age. (D-F) The expression status of the WNT5A gene on GBM, LUSC, and STAD cancer based on sample types and patient’s gender. (G-I) The expression status of the WNT5A gene on GBM, LUSC, and STAD cancer is based on the patient’s race. (J-K) The expression status of the WNT5A gene on LUSC and STAD cancer based on cancer stages. *P < 0.05. **P < 0.01. ***P <0.001. ****P<0.0001. GBM, glioblastoma multiforme; LUSC, lung squamous cell carcinoma; STAD, stomach adenocarcinoma

We found that patients with higher expressions of WNT5A had significantly better OS in BRCA, KIRP, LUSC, READ, THCA, and THYM. In contrast, patients with higher WNT5A expression in BLCA, KIRC, PAAD, PCPG, SARC, and STAD had significantly worse OS (Figure 3). This indicates that WNT5A may play an essential role in the prognosis of BRCA, KIRP, LUSC, READ, THCA, and THYM, whereas it is associated with a poorer prognosis in BLCA, KIRC, PAAD, PCPG, SARC, and STAD.

**Figure 4:**
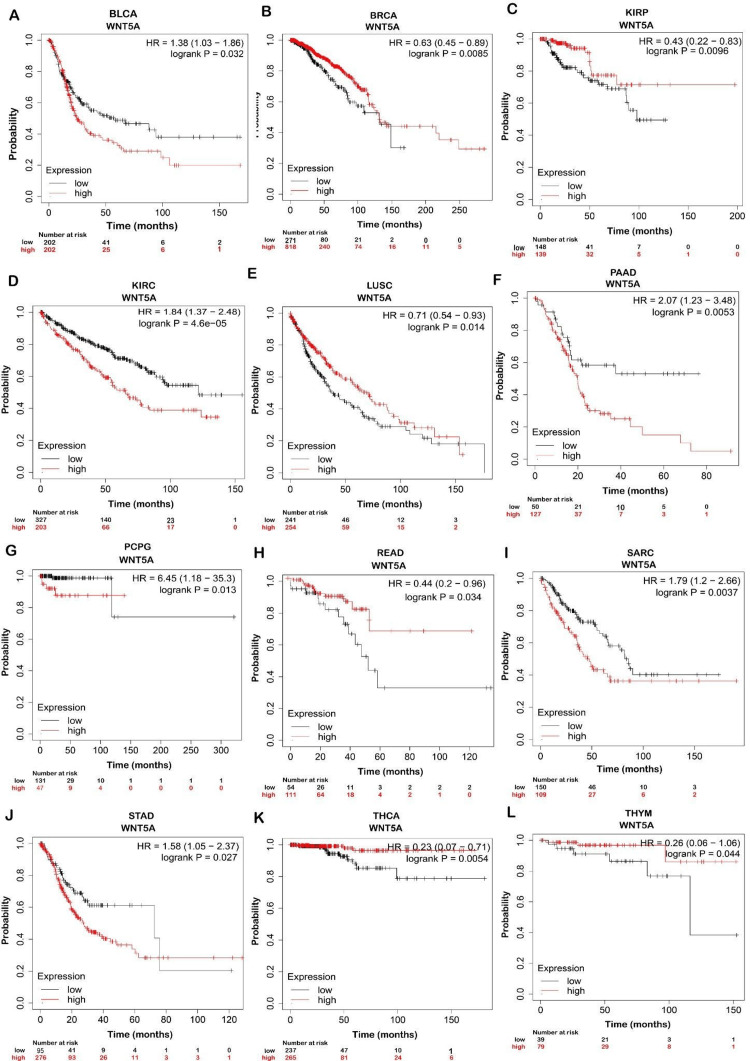
Survival analysis with WNT5A expression in diverse cancer types using the Kaplan-Meier (K-M) database. Overall survival (OS) K-M plots state that WNT5A has a good prognosis in BRCA, KIRP, LUSC, READ, THCA, and THYM, while patients in BLCA, KIRC, PAAD, PCPG, SARC, and STAD. LUSC, lung squamous cell carcinoma; STAD, stomach adenocarcinoma; TCGA, The Cancer Genome Atlas; KIRP, kidney renal papillary; READ, rectum adenocarcinoma; THCA, thyroid carcinoma; THYM, thymoma; KIRC, kidney renal clear cell carcinoma; PAAD, pancreatic adenocarcinoma; PCPG, pheochromocytoma and paraganglioma; SARC, sarcoma; BLCA, bladder urothelial carcinoma

**Figure 5:**
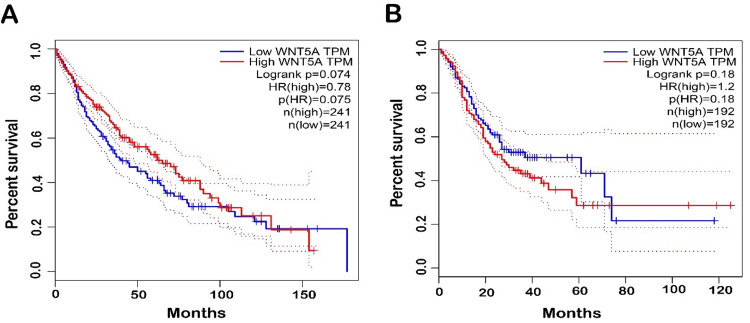
Kaplan graphs illustrate the prognostic value of WNT5A expression in LUSC and STAD cancers in the TCGA database. Time intervals between tumors with greater WNT5A expression levels and those with lower WNT5A expression levels in TCGA tumor types are associated with shorter overall survival time and a poorer prognosis. The red line represents cases with high WNT5A expression, while the blue line represents cases with low WNT5A expression. LUSC, lung squamous cell carcinoma; STAD, stomach adenocarcinoma; TCGA, The Cancer Genome Atlas

Moreover, GEPIA databases were used to assess confirmation of the prognostic significance of WNT5A genes in OS for LUSC and STAD cancer types. Comparisons were made between tumors' OS time with higher and lower levels of WNT5A expression in various TCGA tumor types. The results indicated a shorter OS and poorer prognosis in patients with higher WNT5A expression levels compared to lower levels in STAD. The result can be interpreted as patients with a shorter OS are less likely to live for a specific time. When compared to patients with a better prognosis, those with a worse prognosis are less likely to respond well to treatment and have a higher chance of cancer spreading, coming back, or dying from cancer. Meanwhile, LUSC cancer exhibits better OS (Figure 4). Nevertheless, the analysis revealed no statistically significant association (*P* < 0.05) between the expression of WNT5A and the prognosis of patients with LUSC and STAD in both databases (Figure 5).

Correlation analysis between WNT5A gene expression and infiltrating immune cells

**Figure 6:**
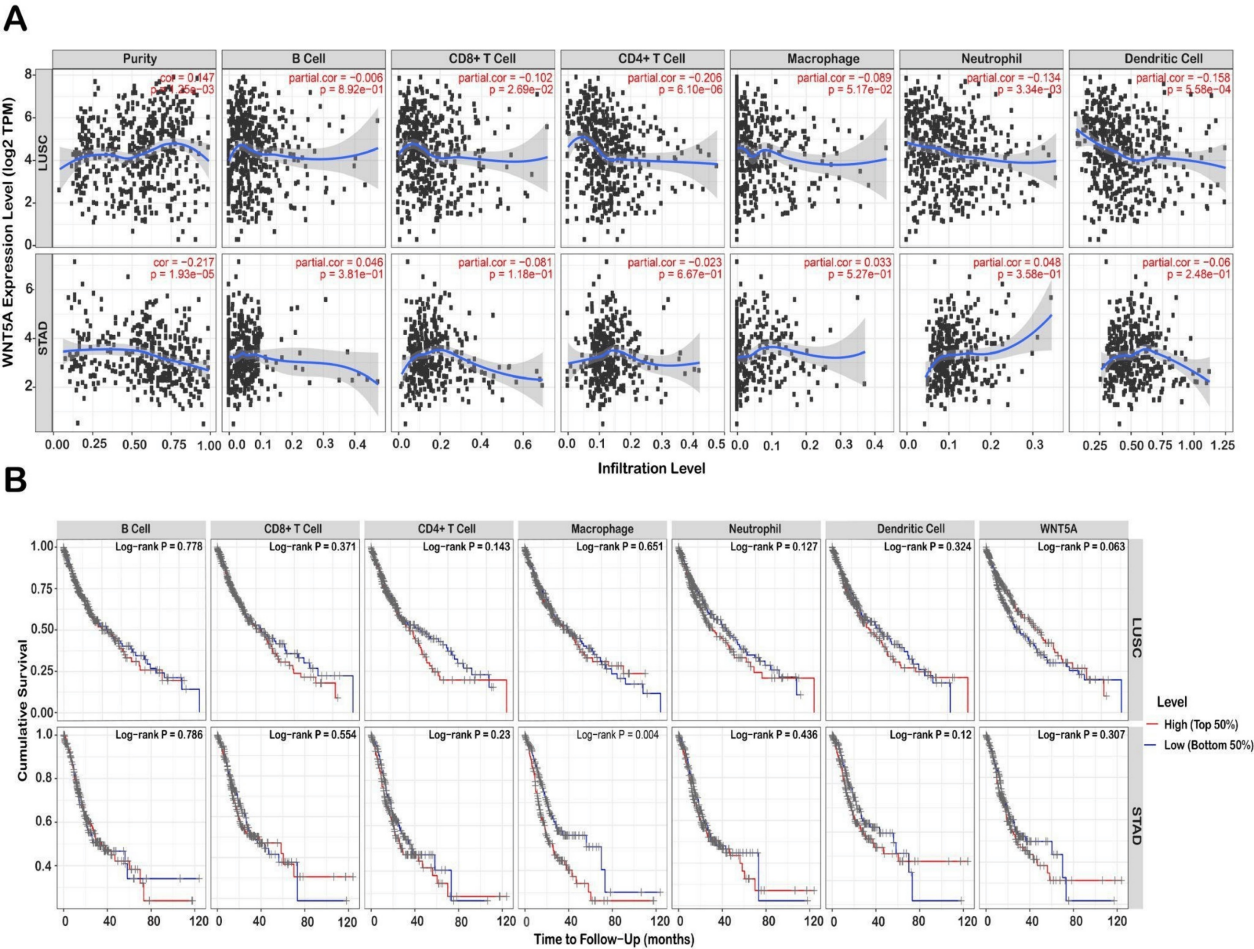
Analysis of the correlation between WNT5A expression and immune cell infiltration level in diverse cancer types. (A) The scatter plots of cancers are generated by one algorithm, including LUSC and STAD. (B) Kaplan-Meier plots for immune infiltrates and genes to visualize the survival differences by one algorithm, including LUSC and STAD. LUSC, lung squamous cell carcinoma; STAD, stomach adenocarcinoma

To uncover variations in the immune environment, the TIMER database examined the associations between WNT5A gene expression and six distinct forms of immunological infiltration, including (cells, CD4+ T cells, CD8+ T cells, macrophages, neutrophils, and dendritic cells). The results demonstrated that the expression levels of WNT5A in LUSC with purity (*r* = 0.147, *P* = 0.001) CD8+ T cell (*r* = -0.101, *P* = 0.026), CD4+ T cell (*r* = -0.205, *P* = 6.10E-06), neutrophil (*r* = -0.134, *P* =0.003), dendritic cell (*r* = - 0.157, *P* = 0.000). Furthermore, the expression of WNT5A in STAD was strongly correlated with the level of purity infiltration (*r* = 0.217, *P* = 1.93E-05) (Figure 6A). In STAD, the *P*-value is significant in all immune cell types, with a positive correlation in B cells, macrophages, and neutrophils and a negative correlation in dendritic cells, CD4+ T cells, and CD8+ T cells. Conversely, in LUAD, survival rates are not statistically significant in dendritic and B cells but are statistically significant in the other immune cells. STAD has a crucial role in immune cells, except for macrophages. These results suggested that the composition of the tumor micro-environment may be a helpful indicator in predicting recurrence and determining treatment strategies in the early stages of development.

WNT5A mutation analysis

**Figure 7:**
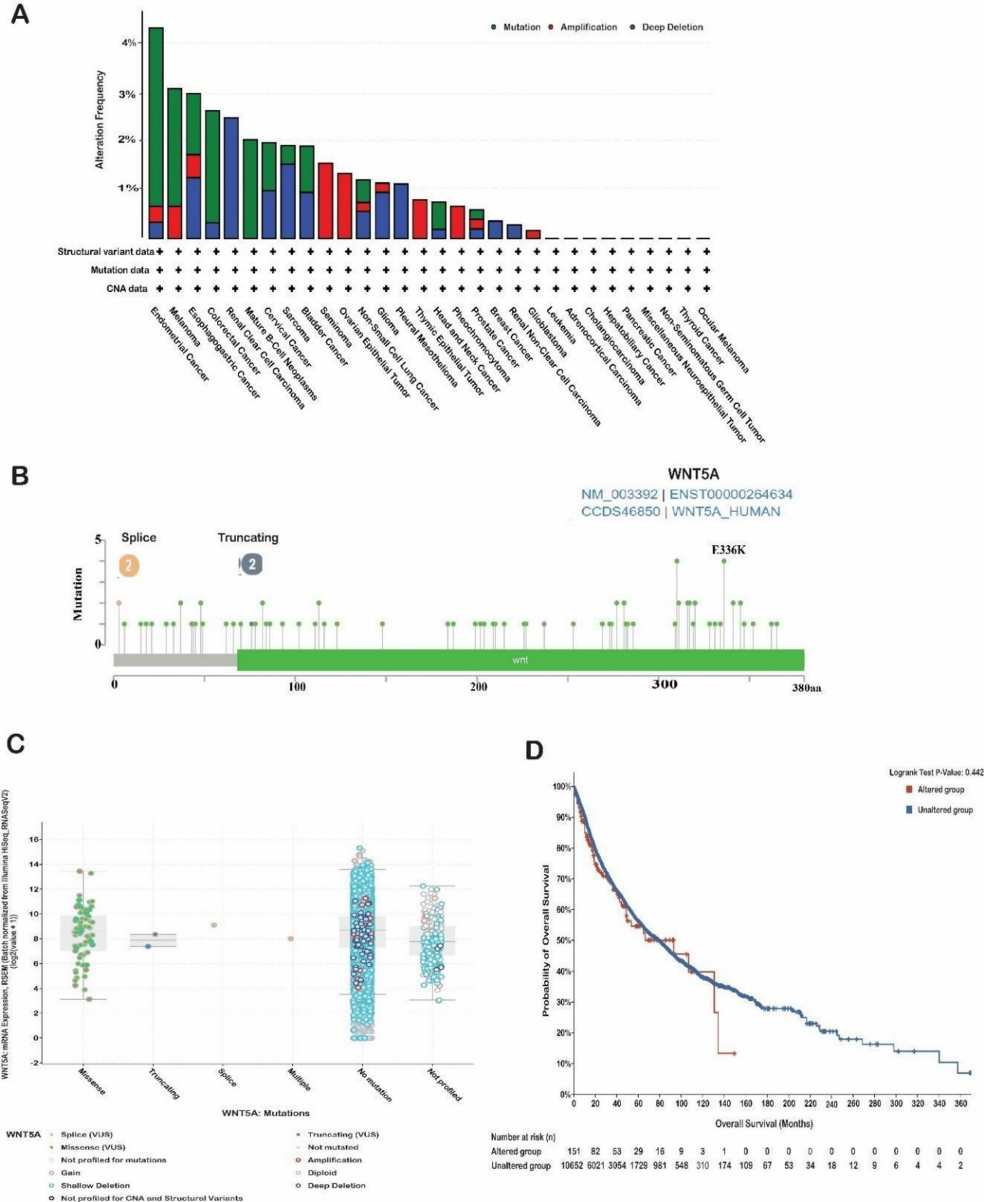
WNT5A genomic alterations in large cancer genomic studies. (A) Genomic alterations in WNT5A were queried using cBioPortal (https://www.cbioportal.org/) containing 10,953 patients in 32 cancer studies. (B) Lollipop plot showing the position of detected WNT5A mutations in the gene sequence. (C) Copy number alteration of WNT5A gene in pan-cancer. TCGA database analyzed the copy number of the WNT5A gene. Gain and amplification were considered as increased copy numbers. (D) The survival rate among patients with various forms of cancer is correlated with genetic changes in WNT5A. TCGA, The Cancer Genome Atlas

We conducted a genetic variation analysis of the WNT5A gene in different types of cancer using the cBioPortal platform. We analyzed the TCGA datasets and found that the WNT5A gene was mutated in 1.4% of the samples we examined. These samples were obtained from 32 different studies, totaling 10,967. The most outstanding WNT5A alterations consist of profound deletions followed by amplification. Our investigation revealed that the most frequent mutations of WNT5A were observed in kidney renal clear cell carcinoma, with a deep deletion frequency of 2.54% (13 cases). The most extraordinary amplification mutation was detected in uterine carcinosarcoma, with a frequency of 1.75% (one case). Notably, no mutations were observed in the expression of WNT5A in diffuse large B-cell lymphoma (Figure 7A). In addition, our findings indicate that the missense mutation for WNT5A is predominant, with position E336K having the highest number of mutations across 10,953 samples for UCEC (10,487 mutation samples), UCEC (727 mutation samples), skin cutaneous melanoma (SKCM, 3,088 mutation samples), and SKCM (2,210 mutation samples) (Figure 7B).

The survival data of patients from various cohorts has been categorized based on the specific cancer type in the WNT5A gene. The study conducted a survival analysis on patients in altered and unaltered groups. Month-based observations of OS have been documented. Based on this study, no statistically significant differences were found between the two groups, as shown by a *P*-value of 0.442 (Figure 7D). The OS of the altered group was 93.01 (95% confidence interval [CI] 48.76-NA), while the unaltered group had an OS of 79.07 (95% CI 73.48-83.93). Subsequently, a more comprehensive examination was conducted to assess the association between the expression level of WNT5A, copy number variation (CNV), and single nucleotide variation (SNV) utilizing the GSCA database. A CNV is a genetic mutation that duplicates a portion of the genome structure, impacting the replication or deletion of a substantial number of base pairs. These copy number alterations differ among people and significantly affect the variance in illness phenotype [15].

**Figure 8:**
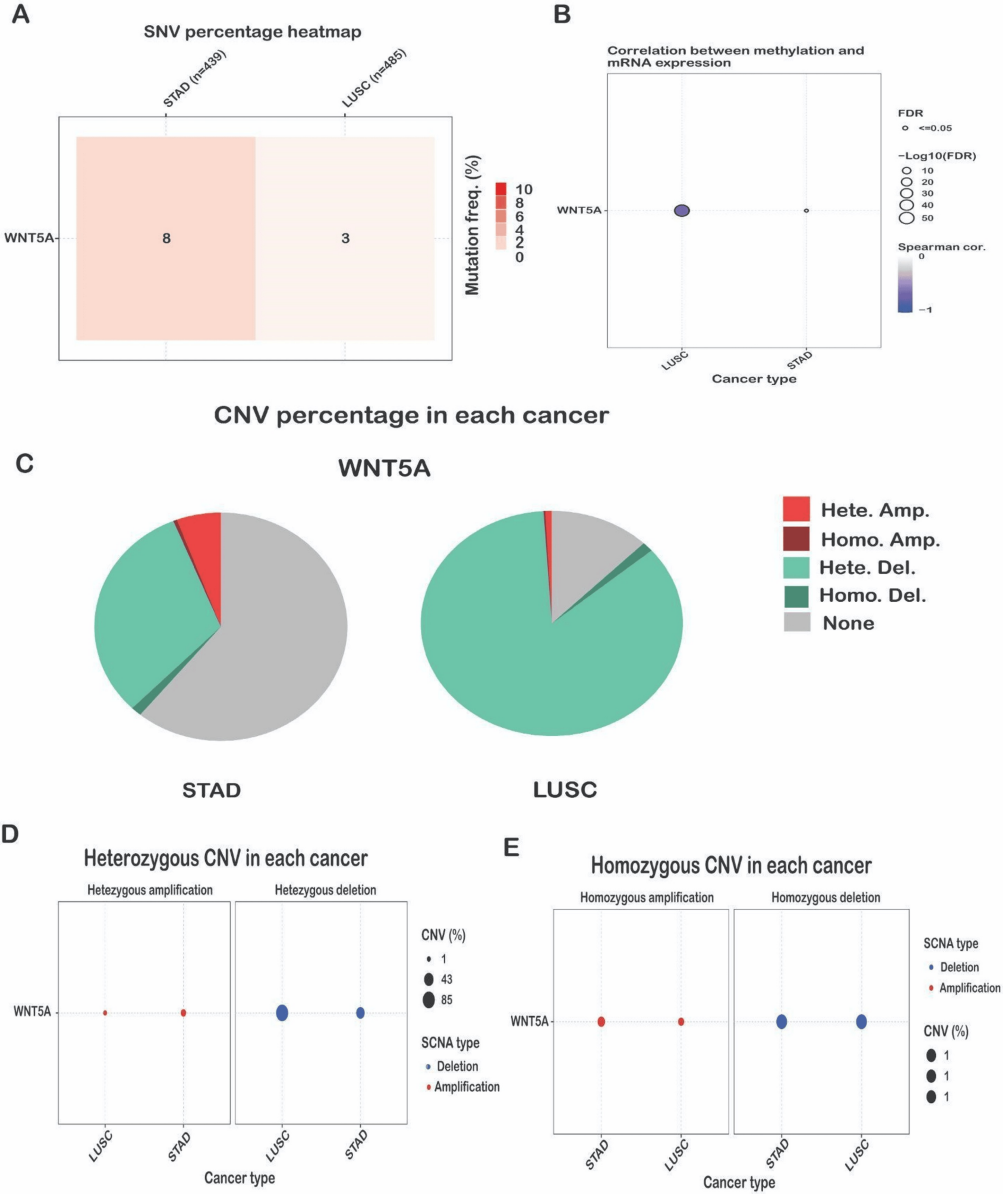
WNT5A gene analysis using GSCA databases. (A) Profile of SNV of the WNT5A gene set in STAD and LUSC cancers. (B) Correlations between methylation and mRNA expression of WNT5A genes in LUSC and STAD cancers. (C) The pie chart depicts the percentage of CNV of WNT5A genes in the STAD and LUSC cancer types. (D) The profile of heterozygous CNV of WNT5A genes in the LUSC and STAD cancer types; bubbles represent the percentage of heterozygous CNV. (E) The profile of homozygous CNV of the WNT5A gene in the STAD and LUSC cancer types; bubbles represent the percentage of homozygous CNV. LUSC, lung squamous cell carcinoma; STAD, stomach adenocarcinoma; CNV, copy number variation

The SNV frequency of WNT5A, as analyzed by the GSCA database, showed that the SNV frequency of WNT5A was high in STAD and LUSC, which was the highest in STAD (Figure 8A). Alterations in DNA methylation play a crucial role in the progression of cancer. Aberrant hypermethylation of the CpG island gene promoter in tumor cells results in transcriptional silence, a characteristic that can persist in daughter cells during cell division [16]. The analysis showed that the methylation level of the WNT5A gene was closely associated with CNV in LUSC (FDR <= 0.0001) cancer type (Figure 8B). STAD showed less correlation when compared to the other two cancer types. The global profile shows the constitution of the heterozygous/homozygous CNV of the WNT5A gene in STAD and LUSC cancer types. The heterozygous deletion CNV is most dominant for all three cancer types, and the highest percentage was found in LUSC (Figure 8C). Heterozygous amplification of CNV is frequent in STAD. It shows a meager percentage in LUSC, homozygous deletion CNV, and homozygous amplification, which does not show any reputation in all two cancer types. Moreover, the heterozygous CNV, including heterozygous amplification and deletion of the WNT5A gene in LUSC and STAD cancer types. The results reveal that heterozygous amplification in STAD is 43%, while in LUSC, it is 1%. Compared to the heterogenous deletion, shown in LUSC at 85%, and STAD at 43% (Figure 8D). Similar to the heterozygous CNV, including the percentage of homozygous amplification and deletion of the WNT5A gene in STAD and LUSC cancer types. The results for both heterozygous CNVs reveal 1% CNV (Figure 8E). These data suggest that WNT5A has a genomic mutation in various types of tumor tissues and may be involved in the occurrence and development of tumors.

## Discussion

The prognosis of cancer patients has improved as immunotherapy and targeted therapies have become more widely used [17]. However, because of the variety of cancer patients, their overall survival remains dismal [17,18]. As a result, researchers are increasingly interested in finding new therapeutic targets for immunotherapy. From another perspective, a pan-cancer study can provide comprehensive insights into the significance of a gene from many aspects in numerous cancers through mining significant databases, which is a helpful way to look for promising targets for tumor therapy [19].

We used the GEPIA, TIMER, and UALCAN datasets to investigate the expression pattern of WNT5A across diverse malignancies, hoping to find substantial changes in WNT5A expression between normal and malignant samples. According to the TIMER database, WNT5A is considerably higher in LUSC and STAD than in standard samples. Upregulation of WNT5A in LUSC suggests a role in disease development [20,21]. WNT5A, a nonclassical WNT signal molecule, is largely conserved across species and is essential in embryonic development, clinical diseases, and internal environmental homeostasis [16]. WNT5A plays a crucial function in embryonic development, with high expression levels in many organs and tissues during the embryonic stage but decreasing in adult tissues [22,23]. Evidence has shown that the expression of WNT5A is upregulated in response to pathogen exposure in immune cells [24]. Interestingly, our GEPIA and TIMER public databases data revealed that WNT5A was more highly expressed in LUSC and STAD. Microorganisms, including bacteria and viruses, can cause inflammation and immune cell activation [25,26]. Hence, it is reasonable to hypothesize that the elevated expression of WNT5A could potentially be associated with the infiltration of inflammatory cells and the aggregation of immune cells.

The expression levels of WNT5A were higher in LUSC and STAD samples than normal samples in GEPIA. The UALCAN study observed that the mRNA expression levels of WNT5A were higher in LUSC and STAD tissues compared to normal tissues. This finding aligns with the results obtained from the TIMER database. Ensuring coherence among the three databases for LUSC and STAD has been demonstrated.

We used UALCAN to study the correlation between WNT5A expression and clinicopathological factors such as age, gender, race, and cancer stage for LUSC and STAD. WNT5A expression levels in LUSC and STAD were considerably higher than in standard samples across age, gender, race, and cancer stage. Intriguing findings have been discovered by a research study that examined the correlation between WNT5A expression and age in LUSC. The study revealed a tendency for WNT5A expression to be lower in normal lung tissue across various age groups. This observation indicates a constant trend of WNT5A downregulation in the setting of lower LUSC development, regardless of age.

In diverse TCGA tumor types, the OS time was compared to tumors with higher and lower levels of WNT5A expression. The results suggested a shorter OS and poorer prognosis in patients with higher WNT5A expression levels compared to lower levels in STAD and LUSC. A bioinformatic meta-analysis found that WNT5A is strongly expressed in 617 out of 1,034 gastric cancer patients. These findings suggest that higher levels of WNT5A expression are associated with more aggressive, invasive, and metastatic gastric cancers, leading to poorer OS outcomes for patients [21].

Hence, our study's critical aspect is emphasizing the role of WNT5A in immune cell infiltration and immune escape in different cancers. We investigated the correlations between WNT5A gene expression and six various types of immunological infiltration, including cells, CD4+ T cells, CD8+ T cells, macrophages, neutrophils, and dendritic cells. STAD correlates positively with B cells, macrophages, and neutrophils and negatively with dendritic cells, CD4+ T cells, and CD8+ T cells. Moreover, WNT5A expression in LUSC exhibits a negative correlation in all immune cells. WNT5A expression has been reported to be favorably linked with immune infiltration, stromal scoring, and immunological checkpoints in most malignancies. This shows that WNT5A may be essential in controlling the tumor immune microenvironment [21]. Specifically, STAD WNT5A expression positively correlates with infiltration of B cells, macrophages, and neutrophils but negatively correlates with infiltration of dendritic cells, CD4+ T cells, and CD8+ T cells [21]. A negative connection was observed between the expression of WNT5A and the infiltration of all immune cell types tested in LUSC [21]. These results imply that WNT5A may promote an immunosuppressive tumor microenvironment by facilitating the recruitment and function of specific immune-suppressive cell types while reducing the infiltration and activity of anti-tumor immune cells [21,27-29].

Subsequently, we used the cBioPortal platform to analyze genetic variation in the WNT5A gene in various forms of cancer. The most notable WNT5A changes are significant deletions followed by amplification. According to one study, the most important genetic alterations in WNT5A across diverse cancers are deletions, followed by amplifications [30]. Moreover, The SNV frequency of WNT5A, given by the GSCA database, was high in STAD and LUSC. The heterozygous deletion CNV is most dominant in the STAD and LUSC cancer types. These results align with a study by Feng et al. who found that WNT5A has a high frequency of SNVs in STAD and LUSC, with heterozygous deletion CNV being the most common genetic alteration [21]. The findings showed that WNT5A experiences significant genomic changes across various cancer types, such as deletions and amplifications. These genetic alterations appear particularly widespread in STAD and LUSC, where WNT5A has a high SNV frequency and is frequently affected by heterozygous deletions. More study is needed to fully understand the functional repercussions of these WNT5A genetic changes and their impact on tumor biology and the immune microenvironment.

Depending on the biological environment and receptor expression, WNT5A can have tumor-suppressive and tumor-promoting actions. It can activate or inhibit the WNT/β-catenin signaling pathway in a receptor-dependent manner [22,31]. This can produce inconsistent conclusions about the role of WNT5A in various cancer types. The mechanisms by which WNT5A can influence cancer development are highly context-dependent and involve the modulation of different signaling pathways, cellular behaviors, and the tumor microenvironment. The specific effects of WNT5A can be both tumor-suppressive and tumor-promoting, highlighting the need for a deeper understanding of the complex role of this signaling pathway in cancer.

The limitations of this study are that it relied on computational analysis for a public dataset. So, wet lab analysis is needed for more validation of these results and to identify how WNT5A expression affects the survival outcome of patients with STADA and LUSC as well as its correlation with immune infiltration.

## Conclusions

The present study is the initial pan-cancer examination of WNT5A. It is a comprehensive assessment of the expression of WNT5A in different types of malignancies, aiming to elucidate its function and its impact on the advancement of cancer. The findings of this research contribute valuable knowledge for future studies, namely, in early cancer detection and the detection of genetic modifications in WNT5A within patients. Our findings provide a foundation for investigating the relationship between WNT5A expression and the proportion of immune cells in different types of cancer, particularly in STAD and LUSC.
